# Involvement of charities in Iran’s health care system: a qualitative study on problems and executive/legal/supportive requirements

**DOI:** 10.1186/s12913-021-06187-9

**Published:** 2021-02-25

**Authors:** Raana Gholamzadeh Nikjoo, Yegane Partovi, Nasrin Joudyian

**Affiliations:** 1 Department of Health Policy and Health Services Management, Tabriz Health Service Management Research Center, School of Management and Medical Informatics, Tabriz, Iran; 2 Department of Health Policy and Health Services Management, School of Management and Medical Informatics, Tabriz University of Medical Sciences, Tabriz, Iran

**Keywords:** Charity, Non-governmental organization, Legal requirements, Executive requirements, Supportive requirements, Health care system

## Abstract

**Background:**

This study aimed to reflect on scientific experts’ and executive stakeholders’ opinions on how charitable organizations can participate in the health care system properly and cope with problems, challenges, strategies, and executive requirements at three major levels of prevention, treatment, and rehabilitation.

**Methods:**

A total number of 20 semi-structured interviews were conducted with scientific experts and executive stakeholders, selected for this qualitative study, based on an interview guide. Using the purposeful sampling method, we selected scientific experts with 5 years of experience in the health care system and executive stakeholders who had 5 years of experience in charitable activities. We applied a framework method for data analysis, and the main themes were extracted through MAXQDA software.

**Results:**

Our findings revealed that charitable organizations at the major levels of the health care system, i.e., prevention, treatment, and rehabilitation, possessed the necessary capacities to provide services effectively. Nevertheless, charities encountered some problems, e.g., financial instability, non-recognition of donors’ legal status, non-involvement in policy-making, inadequate cooperation from other agencies, absence of transparent programs and goals, together with weaknesses in advertising and attracting donations. It was noted that the government should take more operational steps towards supporting such organizations, e.g., by granting special facilities and exemptions, engaging charities in policy-making and training processes, and empowering them in terms of the production of resources. Charitable organizations are also recommended to establish external communications with other bodies such as municipalities, secretaries of state, governorate offices, welfare organizations, relief committees, and medical sciences universities.

**Conclusions:**

Charitable organizations have the potentials to provide health care services at prevention, treatment, and rehabilitation levels. Thus, it is of utmost importance to adopt strategies such as creating sustainable funding sources, training charity managers with a wide variety of scientific management techniques, and implementing their intellectual capacities in legislative and planning processes.

## Background

Accurate and responsible management of community health is regarded as one of the vital criteria and performance indicators of a public health system. Since health promotion is among national priorities and the governments’ primary responsibility, its implementation requires a continuous creation of a vision, illumination of health care policies, orientations,, and legislations, and the establishment of intersectoral cooperation between all relevant and irrelevant organizations and supporting them. Moreover, there is a dire need to set up a secure and reliable information system, mainly affiliated with the Ministry of Health and Medical Education [1]. Notwithstanding, the public sector in all countries has failed to provide health care services to the community for various reasons such as insufficient infrastructure, limited funding and resources, long administrative structure and bureaucracies, political instabilities, inefficient and unskilled workforce, low quality of services, and discriminatory distribution of government resources and facilities, especially in provincial capitals. In developing nations, health status is also poorly evaluated due to the lack of access to health care services attributable to financial, geographical, physical, and social reasons along with severe poverty, low awareness and health literacy within society, and inequalities in receiving such services [2, 3].

Since the mid-1970s, non-governmental organizations (NGOs) have significantly multiplied in developing and developed countries. The governments worldwide, particularly in developing nations, have considered NGOs the most appropriate way to achieve maximum population coverage for health care services. Accordingly, such organizations can perform innovative activities, attract public participation, especially in the disadvantaged and rural communities, and empower populations (who live in weakest strata of society) by helping them to improve their quality of life [4–6]. Studies have thus far revealed that NGOs’ duties in the health sector are not the same in each country, and it depends on some specific characteristics of each region, including levels of development, institutional framework, cultures and traditions, resources, and other needs. Investigations regarding activities fulfilled by NGOs in various countries have further demonstrated that such organizations simply provide health care services to the public in nine categories, i.e., hospital services (viz. general, specialized, and psychological), outpatient/mobile services, rehabilitation and long-term services, and supportive and diagnostic services (namely, imaging), and prevention services (mother-child, occupational health, school, family, prevention programs for diseases, and other public health services).

Moreover, delivery of medical goods, health research, financing, and health system management have been introduced as other services [7, 8]. At the heart of civil society, NGOs have been similarly recognized as the most important and the best tools of organizing public participation. As non-profit volunteer organizations, NGOs are also at the service of people, so charities are called “the voice of the people” [9]. In Iran, the number of these organizations has also grown from 2000 cases in 1997 to about 10,250 in 2005 and exceeded 17,000 charities in 2011. Further, Articles 95, 97, 104, 140, and 163 of the Law of the Fourth Economic, Social, and Cultural Development Plan of Iran and the Horizon of Iran 1404 have been enacted in support of public organizations and charities. The government’s sensitivity has been directed towards the activities of these charitable organizations that should be managed at all major levels of prevention, treatment, and rehabilitation [10]. Studies in Iran have further established that while NGOs are more involved in providing health care services, no NGOs have been able to participate in all four functions of the health care system (namely, stewardship, service delivery, financing, and resource provision). Studies have also suggested that until 2015, 40,000 and 289 billion rials of charitable donations to the health sector in Iran have been related to constructing and equipping hospitals and expanding health care service delivery infrastructure. However, such hospitals have failed to operate and have ultimately shut down because no attention has been paid to their future financial resources. Charitable contributions to universities of medical sciences in Iran were 15,000 million rials in 2016 and about 17,000 million rials in 2017. Therefore, instead of supporting the government to meet the health needs of the public, they have added more problems to the ones that the health care system faced [1, 11].

Considering the non-clarity of activities and involvement of NGOs in achieving the goals of Iran’s health care system and the associated problems, and with regard to the need for the exact information to plan and create policies in the health care system, we conducted the present study to investigate the views of scientific experts and executive stakeholders on proper participation, problems, challenges, and strategies, along with executive requirements of charities at three major levels of prevention, treatment, and rehabilitation.

## Methods

The present study was a qualitative one. Using the purposeful sampling method, we recruited the study sample. A total number of 20 semi-structured interviews were conducted with scientific experts and executive stakeholders, including ten executive stakeholders from charitable organizations (three in the field of prevention, four in the field of treatment, and three in the field of rehabilitation). The other ten experts were individuals from the State Welfare Organization of Iran, the Social Security Organization, and the Association of Health Donors, faculty members as scientific experts in this field, researchers, scholars, experts, and heads of the Charitable Activities Departments of medical sciences universities. Recognition of NGOs as executive stakeholders was done through Iran’s Charity Data Portal and a list of charitable services. After the recognition process, we prepared information for three major levels of prevention, treatment, and rehabilitation separately. With regard to the research purpose, charities that had directly operated at these levels became the base for the study, but the ones that were indirectly involved in health care services were excluded. The inclusion criteria were having 5 years of experience in the health care system for the scientific experts and the same experience in charitable activities for executive stakeholders, and a willingness to cooperate in the interviews. The interview guide was also designed with reference to the review of the literature on this subject matter, and two in-depth interviews were done at the onset of the study. The interviews were focused on proper participation, problems and challenges, strategies, and executive requirements of charities at all prevention, treatment, and rehabilitation levels. The interviews lasted 20 min on average and were performed upon request of time and at the workplace of the interviewees or via phone contacts with organizations and experts working in other provinces. Depending on the interviewees’ experiences and knowledge, purposeful and relevant questions were raised to get detailed and practical information. For example, if an interviewee had additional information about the main source of financing, along with asking other questions delineated in the interview guide, more efforts were made to pose more probing questions about the source of funding to obtain a more precise picture.

The data were analyzed through the framework method. The five-stage process of data analysis, i.e., understanding (specifically, familiarization), identifying a thematic framework, coding (viz. indexing), charting and mapping, and interpreting, was further performed. The main themes were then extracted using MAXQDA software. Upon obtaining the study participants’ consent, the content of the interviews was recorded and transcribed verbatim. As the interviewer received duplicate comments, she stopped data collection and started data analysis. In other words, the interviews lasted until data saturation reached, a point at which “no additional data were being retrieved”. The transcribed content was subsequently matched with the notes taken during the interviews, and conclusively a single text was prepared. In addition, the participants’ views were read several times to gain a general understanding of the texts and to reach immersion. The full texts were successively read line by line. The concepts and the features proposed were also extracted from the opinions, which were then delineated and assigned ‘codes’ or names that best captured the essence of the themes or the issues identified. The interview guide as a starting point for creating overarching categories and any emergent themes from the transcripts were coded in the form of responses to each question. Afterward, the themes were analyzed at the next stages, and the identified cores were retrieved as scientific phrases and classified into main themes and sub-themes based on the proximity of the concepts [12].

This procedure was led independently by two researchers as members of the research team and finally implemented during a meeting through discussions and resolution of disputes. To enhance the consistency and the accuracy of the results, four criteria of credibility, confirmability, dependability, and transferability, developed by Lincoln and Guba, were utilized. To boost the credibility and confirmability, immersion and peer reviews, expert opinions, and participants’ reviews were employed. Thus, after the meetings and following the recapitulation of the views, a summary of the extracted statements was submitted to the participants based on the notes taken during the meetings, so that the erroneous and ambiguous cases could be corrected. To confirm and resolve possible discrepancies, the interview texts were also provided to the interviewees. For dependability, two members were additionally recruited for coding. Finally, expert’s opinions and the purposeful sampling method based on inconsistency were utilized to meet the criterion of transferability [13].

## Results

In the present study, ten charitable organizations as executive stakeholders in the fields of heart diseases, diabetes, hemophilia, thalassemia, multiple sclerosis (MS), celiac disease, specific diseases, mental diseases, and Down’s syndrome at three major levels of prevention, treatment, and rehabilitation were included. Moreover, ten experts including one member from the State Welfare Organization of Iran, three members from the Social Security Organization affiliated to the Ministry of Health and Medical Education, two members from the Association of Health Donors, two faculty members, one expert researcher, and two experts and heads of the Charitable Activities Departments of the universities of medical sciences, were recruited in this study (Table 1). Regarding the thematic analysis, ten main themes and 66 sub-themes were identified, as described below. Besides, each concept was summarized, as depicted in Table 2.

**Table 1:** Baseline characteristics of study participants

Characteristics		Number	Percentage
Gender	Male	15	75
	Female	5	25
Job position			
Executive stakeholders	Heart diseases	2	10
	Diabetes	1	5
	Hemophilia	1	5
	Thalassemia	1	5
	Multiple sclerosis (MS)	1	5
	Celiac disease	1	5
	Specific diseases	1	5
	Mental diseases	1	5
	Down’s syndrome	1	5
Head of unit		2	10
Expert of unit		5	25
Researcher and faculty member		3	15
Years of experience in current job	5–10	6	30
	10–15	7	35
	>15	7	35

**Table 2:** the sub-themes of involvement of charities

Main themes	Sub-themes
Main objectives of charities in the health care system	- Helping the poor and the disadvantaged - Preventing diseases, promoting education, and expanding empowerment
Services at prevention level	- Screening for communicable/non-communicable diseases - Training - Disseminating health-oriented information - Equipping and developing infrastructure in health care centers - Participating in health-related research - Training health educators - Providing counseling and supportive services - Distributing food supplements and preventing malnutrition-related diseases - Supplying health-hygiene tools
Services at treatment level	- Offering pharmaceutical services - Delivering a wide range of treatment services for all types of diseases - Manufacturingknowledge-based products - Arranging for home care support services - Purchasing medical equipment - Constructing hospitals - Covering expenditures - Giving support services in an indirect manner
Services at rehabilitation level	- Implementing physiotherapy, speech therapy, and occupational therapy - Providing financial support for rehabilitation services
Legal/executive/supportive requirements for charitable activities	- Discovering causes of diseases and health risk factors by the Ministry of - Health and Medical Education - Introducing areas in need of more involvement - Developing main strategies to get charities engaged in planning and policy-making - Holding educational courses and programs specially in the field of management and empowerment - Granting special taxes and legacy exemptions - Helping charities in attracting multi-source financial resources and selling their products and services - Giving practical motivation to charities by the government
Main problems facing health charities	- Neglecting charities by the government and having poor faith in these organizations - Financing in an unsustainable manner - Giving insufficient financial aids to people - Doing parallel works - Having inadequate cooperation between governmental/non-governmental agencies and donors - Having inadequate public participation due to weaknesses in advertising and attracting charitable donations - Lacking a legal status for donors - Having no involvement in policy-making and planning - Encountering exhausting executive processes - Having no clear goals and policies - Not having public awareness towards purposeful aids in the health care system
Suggested strategies	- Contributingby the government through programs and interventions in financial affairs of charities - Granting special taxes and legacy exemptions - Setting up endowment funds - Fulfilling scientific management - Taking over responsibilities for supervising and organizing charities by separate organizations independent of the Ministry of Health and Medical Education - Giving responsibilities to charities - Asking charities to play a partin health-related decisions and policies - Holding various meetings to have appropriate interactions between universities and donors - Gaining donors’ trust
Main sources of financing charities	- Donating from the public - Generating income by charities - Granting by the government in the form of special budget lines - Paying membership fees by clients
Extra-organizational communications of charities	- Municipality - Governorate office - State Welfare Organization of Iran - Universities of medical sciences - Pharmaceutical companies - Insurance companies - Ministry of Education - Endowments and Charity Affairs Organization
How to coordinate activities of charities and other parts of the health care system	- Establishing a relationship between insurance companies and the Ministry of Health and Medical Education - Making governmental managers informed of challenges as well as financing and managerial status of charities - Establishing a constructive relationship - Having interactions but no competitions - Using ideas raised by charitable organizations in health-related decisions and policies

### Goals of charitable activities

This theme was the first one identified about the goals and the philosophy of charities in Iran. The participants mainly acknowledged that such organizations were to lend a hand to people in need, promote education, and increase preventive measures. In this respect, Participant No.2 said that “*As you know, the main goal of charitable organizations is to provide support to people who cannot afford it.*” Participant No.5 also stated that “*For example, the main objective of our organization is to help the poor and the needy, especially the ones having no means to pay high costs of multiple sclerosis.*” Also, Participant No.4 added that, “*I think, enriching physical and mental development, boosting individual and social conditions, as well as promoting children’s health status in all aspects, particularly delivery of health and medical counseling to their parents, referrals to medical centers, prevention, and rehabilitation are among the goals of this organization*.”

In addition to providing prevention services, such health-related organizations were also able to deliver services in treatment and rehabilitation. According to Participant No.1, “*Objectives can actually vary everywhere and include a wide range of interventions. Some organizations may be merely treating specific diseases, some may simply focus on prevention services, and others may put emphasis on rehabilitation, while some may fulfill all of them together*.” Besides, Participant No.6 reiterated that “*The main goals of such organizations are health education and promotion within societies*.” Participant No.8 correspondingly said that “*The overall goal of charities is to help many deprived people who are not able to live a normal life like others. For example, our organization empowers cancer patients to rebuild their lives*.” In addition, Participant No.7 restated that, “*If you ask me, life is the right of all human beings. Now, for some reason, some people are deprived of life filled with relative peace and welfare, and the government cannot aid all people. They can make a relatively satisfying life for such people, especially now that they are sick*.”

### Services at Prevention Level

In this regard, the participants provided information on how charitable organizations could get involved and what services were best done at the prevention level. One thing that charities could do to help in terms of prevention was to educate and promote community health. Commenting on this issue, Participant No.3 declared that, “*Most donors in our country are working more in the field of treatment. The field of prevention is a little smaller. In my idea, a lot can be done in the field of health education. There are also numerous organizations working directly in this field.”* Participant No.2 also said, “*Of course, there are more discussions about prevention in developed countries. I myself have seen several times in these countries that a series of campaigns have run to educate and promote health regarding many diseases and to raise public awareness. What is wrong with our charities going this way?*” As well, Participant No.9 stated that “*It is possible to start from the same mosques, to make the best use of the public themselves, and to carry out a series of health-oriented educational activities in the form of neighborhood groups that are funded by the donors themselves. The capacity of charities can also be exploited to distribute food supplements and to prevent diseases caused by malnutrition*.” According to Participant No.11, “*Unfortunately, nutritional status and lifestyle are currently very poor. Now, wealthy people can make up for their nutritional deficiencies, but poor families cannot afford it. I think vitamin D and iron supplements can be given, especially at schools*.” Besides, Participant No.7 added that “*I do not know about the mechanisms by which children suffering from malnutrition or affected with nutritional problems can be identified and benefit the right level of nutrition by giving supplements or meals.*”

Other preventive measures taken by charities were the involvement of volunteer physicians, nurses, paramedics in screening, counseling, and other support programs and the distribution of health tools. For example, Participant No.8 put it in this way, “*Some activities do not cost that much. Through establishing health stations in marginalized and poor areas, measuring blood sugar, blood pressure, height, and weight, as well as providing a series of counseling services for expecting mothers or family planning or nutrition issues can be easily delivered, even by medical, nursing, and midwifery students.*” Considering the distribution of health and hygiene tools by institutions, Participant No.10 said that, “*It is possible to distribute a series of health items such as condoms and build toilets in disadvantaged areas and villages, in the form of volunteer groups, with the help of donors*.” Charitable organizations were also able to play a part in equipping, developing, and rebuilding the infrastructure of community health centers and health homes. In this line, Participant No.5 stated that “*Jihadi camps that are today being held could be easily exploited to rebuild community health centers.*” One of the potentials of charities could be thus conducting research, mainly health-related investigations. These organizations could similarly play an important role in the production of medical sciences in health-related projects in cooperation with the Ministry of Health and Medical Education and universities of medical sciences. Accordingly, Participant No.12 reiterated that “*During my travels to different countries, I found out that charities had their own research lines. For example, an organization offering cancer services had also set up a research group to conduct a series of studies on treatment, prevention, or the latest anti-cancer techniques. In our country, as far as I can see, there is no such attitude, or it is very weak*.” It was also possible to promote the culture of prevention, information-giving, and public awareness in the field of health promotion via training courses and programs for health educators provided by charitable organizations. As Participant No.1 put it, “*I started with a series of training programs in this organization. I also designed a set of programs through them. Now, I am educating a trainer to teach people how to prevent AIDS.*”

### Services at Treatment Level

In this section, the study participants talked about how to contribute and what services were best done at the treatment level. One of the main areas highlighted in this respect was hospital construction following demands raised in society, its high efficiency, and impressive effects. Regarding this issue, Participant No.13 stated that “*In our country, most of the donors are working in the field of hospital construction. In the realm of health, the first thing that comes to mind is to build a hospital. Now, I wonder what problems will arise later.*” Besides, donors involved in the treatment field could provide and pay a part or all of medical and treatment costs. For example, Participant No.2 said that “*There is much work to be done in the field of treatment. There are even households that cannot afford to provide medicines for their patients, and they cannot even pay for their doctor’s periodic visits. On the other hand, they are very humble and do not like to ask others for help.*”

One of the most influential areas met by charities was home care is the provision of medical services and nursing at home. In this respect, Participants No.11 stated that “*Some volunteer doctors, with the help of our organization, go to the homes of patients, to visit and treat patients who are not able to move or cannot go to medical centers for any reasons. It is an excellent activity indeed that can be accomplished in nursing.*” Participant No.9 also declared that “*I know a cancer institute in the city of Tehran, which provides nursing and medical services to patients at home*.” Concerning prevention, donors could take effective steps in terms of providing and equipping service infrastructure and even purchasing pieces of equipment. For example, Participant No.14 said that “*After consulting with the chancellors and managers of hospitals and other medical centers, it is possible to inform them about the problems and shortcomings of equipment and facilities, and finally to direct donations towards purchasing a series of equipment for these centers and even register them with the name of the donors*.” In addition, Participant No.10 reiterated that “*Many outpatient clinics and health care centers, even those with limited services, have been recently built by donors, who are kind of working in the field of treatment, and sometimes even the donors themselves do the management work*.” Participant No.12 also said that “*Donors in our province have equipped several operating rooms and intensive care units through the universities of medical sciences*.” In recent years, the development of knowledge-based companies has similarly provided many opportunities for the involvement of charities, especially in the field of treatment. For example, Participant No.15 stated that “*There is much to do in the field of health-related inventions and innovations. In many developed countries, knowledge-based companies put the ideas forward, and charitable donations are being used to mass-produce them. Over recent years, knowledge-based companies in our country have fortunately made good progress, and they are well established in the community. Many of the projects are in the field of treatment, but they have reduced to immature ideas due to financial problems, and it would be great if we could direct some donations towards this field*.”

### Services at Rehabilitation Level

In this section, the participants provided information on how to get involved and what services were best done at the rehabilitation level. In this field, charities could provide services via purchasing or financing them in the areas of physiotherapy, speech therapy, and occupational therapy. In this sense, Participant No.4 stated that “*Many organizations provide speech therapy services in collaboration with the universities of medical sciences*.” As well, Participant No.7 said that “*For example, our organization has recently signed contracts with several rehabilitation centers, and we are referring our patients to these centers to receive the related services*.”

### Legal/executive/supportive requirements for charitable activities

The study participants expected the government to appreciate active charities as they were implementing their programs and assign some executive work in the field of health to such organizations so that they could have a share in planning and fulfilling health-related policies. In this regard, Participant No.16 acknowledged that “*There are lots of questions, which conferences or meetings have been so far held to appreciate donors and top charities? or which appreciation plaques have been given to donors? I think lack of acceptance and poor faith in donors by the government has discouraged them*.” Besides, the most important request raised by the experts was to involve charities in health-related policy-making. In this respect, Participant No.15 stated that “*Several laws, circulars, and guidelines have been thus far approved and designed for charities, but there are still more than a few legal gaps. I think charities are valuable capacities that can be exploited very well. I wish we, like other countries, would exploit these capacities in our policy-making*.” Besides, the stakeholders believed that the government needed to empower charitable organizations to attract financial resources and generate income. For example, Participant No.17 said that “*Specialized training courses and programs should be held for the managers of the organizations, particularly in the income-generating area by the organizations themselves*.” The study participants also reiterated that the government should apply special tax exemptions to facilitate operations and inspire such organizations. In this regard, Participant No.10 stated that “*Previously, the government had acknowledged, in a guideline, that charities were exempt from taxes, but it was just a guideline, and they did not implement it well.”*

One of the other requirements facilitating such charitable organizations’ activities was to identify the disease’s burden. This area requires the involvement of charities and their access to the information, which can be granted by the Ministry of Health and Medical Education. In this regard, Participant No.8 said that, “*In the field of treatment, we must first pay much attention to existing information about diseases and the burden of diseases in Iran to see which one is a priority. We also need to know which diseases impose more costs on families. We then should plan for them whether in the field of drug supply, prevention, or treatment.*”

### Main problems facing health charities

The most significant problems and challenges discussed in this section were associated with the government’s role and its low faith in the activities of charities working in the field of health. As Participant No.18 put it, “*The government’s role in supporting and financing is minimal*” One of the most significant problems facing all charities was sustainable financing for these organizations. For example, Participant No.9 stated that “*The government does not act very well in terms of funding. The majority of people are merely helping in this way*.” Participant No.10 also added that “*If you ask me, our main problem is how to fund these organizations*.”

Other problems drawn against these charitable organizations were associated with legal gaps in their activities and involvement. In this regard, Participant No.11 stated that “*The government does not care about us at all. We have no legal status. If our legal status had been recognized, most financial problems would be automatically resolved*.” Participant No.6 also said that “*Everything is going on at random, there are no special legal procedures; everything is just parallel work, and no smart coordination is observed*.” Among the barriers to charitable activities was the incomplete implementation of the tax exemption law for charities. In this regard, Participant No.19 stated that “*Instead of taking over the responsibility of providing financial aid to organizations, the government is imposing taxes. Our organization is hardly funded, and I wonder how can it pay such taxes?*” The experts also believed that insufficient constructive and interactive cooperation between governmental/non-governmental agencies and charities was another barrier to these organizations’ activities. For example, Participant No.4 reiterated that “*There should be some kind of interdependence between charities and other government bodies to move forward, not to interfere. I think other organizations view us as rivals, and this culture is wrong*.” Non-involvement of charities in legislation and development of macro-policies, especially in the field of health, could discourage these organizations. In this line, Participant No.7 maintained that “*The health system officials and the government should invite us and inquire about our problems. They need to allow us to attend their decision-making meetings and get involved. They must also let us express our comments and contribute to policy-making. In any case, we represent a large number of deprived people whose voices must be heard*.” In addition, Participant No.10 put it in this way, “*In which meetings and conferences were we invited and asked about our problems and opinions? When there is no one to talk about the problems with this esteemed government, it is we who can bring up the best ideas and speak up and also do the best planning, because we really see the problems and touch them*.” The priority of treatment over prevention was also among the problems facing the health care system, which had affected donors and charities’ activities. Thus, many donors had been attracted to hospital construction and absence of sustainable funding to maintain these hospitals had added one more problem to those challenging the health care system. In this regard, Participant No.20 stated that “*In our society, all donors would like to build a hospital. This shows that there is a problem in building a culture in this domain. The health care system is not just limited to the construction of hospitals. The government must delineate the gaps correctly. I think the government has not publicized problems well. It should be additionally involved in attracting donors to other areas of the health care system, not merely hospital construction*.” Besides, Participant No.3 said that “*Most people would like to build a hospital because of its early return and outstanding effects, while our donors have not been informed that hospitals donated should be funded sustainably. After a while, such hospitals turn into a surplus instead of being helpful. So, one more problem challenges the health care system*.” One other barrier to charitable donations was redundant bureaucracy. As Participant No.12 put it, “*Sometimes, the medical sciences universities unbelievably focus on lengthy administrative work and wrong dos and don’ts on licenses so that donors feel regretful and direct their own donations to other purposes.”*

Moreover, Participant No.18 said that “*One of the departments was supposed to issue a permit for donating several beds to an affiliated hospital, but it was delayed so much that the value of the money dropped and it was not enough to equip those beds. I think this is a great injustice to both people and donors*.” One other problem related to the charitable organizations’ activities was the lack of clear goals and programs to continue activities so that Participant No.16 added that, “*Many charities are moving away from the goals set in their laws towards other areas. Perhaps the most important weakness in their performance is the members’ confusion. Moreover, there are no organized programs and goals in internal activities*.”

### Suggested strategies

Within this theme, the study participants deliberated several solutions to deal with some weaknesses and problems. Accordingly, they proposed the government’s role in charities’ financial affairs by presenting programs and interventions. In this regard, Participant No.15 stated that “*The law should be in such a way that certain earnings be obtained from high-income individuals (especially from doctors’ salaries) and given to charities.”* Participant No.17 also added that “*To establish coordination between charities and the health care system, the Ministry of Health and Medical Education can be asked to develop a special program for charitable organizations based on the participation and cooperation of this ministry in financing such organizations*.”

Another proposed solution was that separate organizations independent of the Ministry of Health and Medical Education should oversee and organize charities. For example, Participant No.20 reiterated that “*The government should not do everything by itself. An independent government body should be in charge of managing and overseeing charities*.” One further barrier to charitable activities was the non-implementation of laws on financial exemptions of philanthropic organizations. In this line, Participant No.19 said that “*In the past, the government had exempted charities from taxes, but now it is only limited to the guidelines, and they do not implement it well*.” The use of capacities and contributions of charities to health-related decisions and policies was one of the solutions addressed by the participants. They believed that charitable organizations could better transfer problems due to their proximity to society’s deprived and disadvantaged strata. In this regard, Participant No.13 stated that “ *Medical sciences universities can appoint a representative among us to attend all their meetings and decisions*.”

Moreover, Participant No.5 said that “*I think charities are endowed with outstanding capacities that can be exploited well at national, macro, and executive levels should they become more empowered. They are also able to accept some responsibilities*.” The participants similarly found the core of charities’ success in the shadow of scientific management and demanded teaching modern management techniques to all heads and managers of charities. For example, Participant No.10 believed that “*The only thing missing in our country is scientific management. In my view, all our decisions and actions are emotional and fleeting. In my idea, scientific management should be taught in all charities.*” One way to support charities was thus to set up endowment funds. In this regard, Participant No.14 reiterated that “*It is possible to establish public donation funds through donors because people trust them. So, public donations can be collected through these funds*.”

### Main sources of financing charities

This theme reflected on the most critical financial sources to fund charities. One of the most significant sources of funding for such organizations could be public participation in various ways. In this regard, Participant No.17 stated that “*People themselves can be a source of income for us. For example, in our organization, many individuals deposit a fixed amount into the charity’s bank account every month.*” Participant No.3 also added that “*Iranian people almost always give positive responses to public works. They easily dedicate their own income to such organizations*.” As well, Participant No.13 said that “*Charities do not hesitate to help people as much as they are donated. The help provided by these organizations depends on that of charitable organizations*.”

In addition, the participants believed that public donations were ineffective by themselves, and charities need to be empowered to generate income through their own skills and capabilities. In this regard, Participant No.9 believed that “*No matter how many public donations are there, they are still not among sustainable financial resources for such organizations. Public donations are merely giving fish to charities. I think fishing should be taught to these organizations to be operating for a long time. I think they should be empowered to produce their own income because this source of income will be more sustainable*.” Participant No.11 also reiterated that “*In many developed nations, charities have their own sources of income. I mean, they have a large number of manufacturing plants that can be funded if the government or the public do not help*.” Another proposed source of funding for charities was government support in the form of a dedicated budget line. For example, Participant No.2 stated that “*If there is no public assistance, such organizations must be shut down. None of the government’s works is right, let alone addressing charities such as planning or budgeting*.” Moreover, Participant No.1 maintained that “*We are currently residing in an Islamic country. If it is really Islamic, good deeds should be on the government’s frontline; I mean, the government needs to determine specific budget lines for charities for good deeds as for other organizations*.”

### Extra-organizational Communications of Charities

This theme talked about intersectoral communication and cooperation between charitable organizations and governmental/non-governmental agencies. In this line, Participant No.1 stated that “*To take part in intersectoral activities of both governmental and non-governmental agencies, various agreements have been thus far signed with the Endowments and Charity Affairs Organization, Tehran Municipality, Ministry of Education, Ministry of Agriculture Jihad, Ministry of Culture and Islamic Guidance, and Prisons Organization of Iran.*” Also, Participant No.19 said that “*Unquestionably, medical sciences universities help charities that would like to work in the health sector.*” Participant No.14 also added that “*Depending on their activities, charities, whether medical or preventive, will be affiliated with various organizations including pharmaceutical companies, insurance companies, hospitals, and governorate offices*.” Finally, the study participants stated that charities needed to cooperate with the medical sciences universities, insurance companies, municipalities, governorate offices, and the State Welfare Organization of Iran.

### How to coordinate activities of charities and other parts of health care system

This theme reflected on how to establish cooperation and coordination between charities and other parts of the HCS. In this respect, Participant No.17 said that “*Relevant bodies and organizations view charitable organizations as their rivals. There should be a state of interaction and cooperation. We are all working to be of assistance to disadvantaged people*.” Besides, Participant No.20 stated that “*Several heads of charities were once invited to a meeting held by our university, as an opportunity to express their problems and challenges. I think the government should hold such meetings much more*.” Participant No.9 also reiterated that “*Given that there is no transparency in tariffs on health care services, the relationship between insurance companies and the Ministry of Health and Medical Education can make it clearer*.” According to the participants, the Ministry of Health and Medical Education and insurance companies could play an influential role in creating such coordination. They correspondingly suggested that the relationship between charities and other organizations should be grounded on constructive cooperation and interaction but not competitions.

## Discussion

The present study aimed to investigate the opinions of scientific experts and executive stakeholders on the proper involvement of charities at three major levels of prevention, treatment, and rehabilitation. Our results showed that from the experts’ perspective, the main objectives of charities in the health care system were to help the poor and the disadvantaged, prevent diseases, promote education, and expand empowerment. It was also natural that charitable organizations could play an influential role in monitoring, attracting public participation and support, promoting health status, and empowerment due to their close relationships with the community [14, 15].

Besides prevention, treatment, and rehabilitation levels, NGOs and charities could get involved operationally provide services. Some services could be further provided and managed by charities directly through screening for communicable/non-communicable diseases, disseminating health-oriented information, equipping and developing health care centers’ infrastructure, contributing to health-related research, training health educators, or indirectly through counseling and support services in the field of prevention. Utilizing donors’ capacities in medical science research to discover the causes and methods of disease prevention, therapeutic and diagnostic methods for diseases, and discovery of new drugs would appropriately lead to a national scientific leap in the field of medical sciences. Concerning treatment, they also had the potential to provide services through follow-ups and a wide range of pharmaceutical, outpatient/inpatient, and diagnostic services directly via covering drug and medical costs or providing home care support services indirectly. However, coverage of expenditures could be fulfilled either directly by organizations such as the State Welfare Organization of Iran and the Relief Committee or indirectly through contracting with medical centers to pay for parts of them. Charities could further provide some structural services such as hospital construction, supply of medical equipment, and manufacture of knowledge-based products in the field of treatment. Given the prevailing culture of the national health care system, i.e., the predominance of treatment over prevention and the fragmentation of services, it seemed that the provision of medical services was more acceptable due to their early effects. The importance of the issue could become very clear once there was ample evidence that national donors were strongly keen to operate in the hospital construction sector merely owing to its early return nature. It should be reiterated that the use of the capacities of such charities could play an essential role in the formation of the post-discharge care system in order to complete the treatment and recovery process in the form of home care services by physicians and nursing volunteers [9, 16–20].

Charitable organizations could correctly assume a role in this area when the requirements and infrastructure were established and implemented by the government, the Ministry of Health and Medical Education, and other relevant bodies. Managers and policy-makers in the Ministry of Health and Medical Education would be thus able to facilitate the provision of effective and targeted services by donors by identifying disease burden, high-risk groups, and target ones in need of medical and educational interventions. Moreover, they could make the best use of needs assessment and prioritization of health problems, detection, the introduction of areas in need of participation, and disseminating this information by the mass media.

To provide facilities and increase charities’ ability, the government must analyze the financial and managerial capacity of charitable organizations comprehensively and develop policies and programs to pave the way for their involvement. Unfortunately, the findings suggested that inappropriate cooperation of some universities with donors had led to their discouragement and low attendance at the community level. The Ministry of Health and Medical Education and the medical sciences universities need to consider charities as auxiliary arms and establish constructive engagement with them. The government’s capacity is also limited in both funding and services, so the provision of health care services and promotion of community health will not be achieved in the absence of such organizations. Building a relationship through trust and respect will correspondingly result in donors’ satisfaction and the repetition of their charitable activities. Many factors, including the lack of transparency and accountability, were also contributing to the descending trends in philanthropic donations to the health care system. The chancellors of the universities can accordingly motivate and gain the trust and continuity of the cooperation of charitable organizations through transparency and accountability, periodic feedback, and performance reports. It should be noted that trust and openness are bilateral issues demanding special attention from the Ministry of Health and Medical Education, medical sciences universities, and charities. Given that trust is the main capital of charities, some sabotage and irregularities can cause problems for such organizations due to the lack of transparency and failure to gain donors’ trust. Many national charitable organizations fail to gain the confidence of donors and provide sustainable funding due to the lack of specific objectives and non-transparency due to the lack of strategic and short-term operational plans [21–23].

As mentioned earlier, charities were struggling with many problems, some of which arising from environmental and external factors and others in light of their internal conditions. Among the external issues were instability of financial resources, neglect and low faith in charities by the government, no recognition of donors’ legal status, non-involvement of charities in health-related policies and planning, and inadequate cooperation between governmental/non-governmental agencies and donors. The lack of straightforward programs, goals, and performance by charities as well as weaknesses in advertising and attracting charitable donations, were some of the internal problems. The results of the present study demonstrated that the main sources of funding for charities could be public donations, income generated by such organizations, grants by the government in the form of special budget lines, and membership fees paid by clients. The problems in financial resource instability and allocation of a specific budget continues the instability problem since it can lead to charities’ more dependence on the government. To provide sustainable financing, charitable organizations also need to be empowered and take further steps by holding exhibitions, implementing joint projects, creating partnerships with public and private agencies, conducting research projects and obtaining grants in this way, and investing in areas generating value-added. In addition to funding, other issues such as modes of spending, number of clients covered, and types of services are of utmost importance, which can be provided only through an accurate audit system, financial transparency, and social status of these organizations. Unfortunately, charities avoid any risks due to fear of failure to prevent loss of charitable capital caused by possible malfunctions. Therefore, they do not enter into various projects to boost their investment. Achieving this level of capability and overcoming these fears and uncertainties will be thus impossible with regard to improper scientific management by the heads of the charitable organizations themselves. Scientific management will be accordingly one of the critical factors affecting charities’ success in maintaining donors and effective advertising. Familiarizing charity managers with various scientific management techniques, including customer relationship systems, will help establish closer and deeper relationships with donors and ultimately attract sustainable financial resources. With respect to insufficient government support for charities, it could be inferred that such organizations were being ignored by the government policy-makers and planners because they were considered as non-medical organizations with no effects on public health.

On the other hand, one of the most important demands raised by charities from the government was why they were not involved in planning and policy-making. Therefore, the government should provide numerous opportunities for such organizations to bring up their own problems and ideas, make use of advice and opinions of NGOs in specialized meetings, delegate responsibilities to them, and welcome and support their activities since these organizations have the deepest and the closest connections with society, especially the disadvantaged strata. Previous studies in this field had also suggested using the capacities of NGOs in decision-making and respecting their counseling positions [24–30].

Just as charities and NGOs are regarded as the government’s helping hands in providing health care services to the public, they would not be able to survive without intersectoral cooperation and communication with other governmental/non-governmental agencies such as municipalities, secretaries of state, governorate offices, welfare organizations, relief committees, medical sciences universities, and the like. Constructive and non-competitive interactions between charitable organizations and other bodies could thus induce the synergy of resources and facilities, elimination of parallel works, and use of capabilities and mutual capacities to provide desirable services to people in society, and ultimately to control and prevent many social and health-related risks [21, 22, 30].

To better and more efficiently support charities, the government must employ incentives and support mechanisms, including granting special exemptions, empowering and training managers, especially in the areas of sustainable financing and resource production, and using intellectual capacity in legislative processes. Although some support mechanisms such as tax exemptions have not been fully met, inadequate government oversight would allow some businesses to pay lower taxes in the form of stimulation assistance in consultation with some charitable organizations. Thus, it is a good idea to apply the necessary supervision in a precise and transparent manner to the books of accounts of these organizations due to the socio-economic effects of tax exemptions [31].

### Limitations

The present study was not devoid of limitations. Unfortunately, access to some managers and experts followed by canceling the Vice-Chancellor for Social Affairs in the Ministry of Health and Medical Education was difficult. Furthermore, some interviewees were facing constraints in expressing their opinions due to political considerations, institutional positions, or even insufficient knowledge. Moreover, communication with charities was challenging because some information was vague, such as organizations’ names and wrong numbers or addresses registered on Iran’s Charity Data Portal.

## Conclusion

As established by the findings of the present study, charities have the potentials to provide services at all three major levels of prevention, treatment, and rehabilitation. Moreover, all charitable organizations’ main objectives are to help disadvantaged people, prevent diseases, improve education, and boost empowerment. Charities are also facing financial instability, no recognition of donors’ legal status, non-involvement in policy-making and planning, inadequate cooperation between governmental and non-governmental agencies, no transparent programs, goals, and performance, together with weaknesses in advertising and attracting charitable donations. Accordingly, the government should take effective steps to facilitate philanthropic activities through support mechanisms such as granting special facilities and exemptions, involving charities in planning and policy-making, as well as training and empowering them in the production of resources, and so on. Furthermore, charitable organizations should establish extra-organizational communications with other bodies such as municipalities, secretaries of state, governorate offices, welfare organizations, relief committees, universities of medical sciences, etc., to fulfill their missions. Finally, it is noteworthy that the government can easily take advantage of the capacities of charities in producing, disseminating, and raising public awareness regarding health, justifying people to choose appropriate health interventions, conducting health research, and applying their results, as well as changing social attitudes towards health. Besides, charities have the potentials to finance health care services, administer justice in resource allocation, and create accountability and transparency in resource allocation and management. Charitable organizations also represent the interests of the community and the general population in policy-making and can be thus as catalyst processes to increase public support for health policies. As a whole, future studies are suggested to investigate the impact of the involvement of charities and NGOs in the efficiency and effectiveness of the health care system programs, or how NGOs and charities participate in the health care system policy-making and planning following upstream documents.

## Data Availability

The datasets used and/or analysed during the current study available from the corresponding author on reasonable request.

## Abbreviations

NGOs: Non-Governmental Organizations
MS: Multiple Sclerosis
